# The noncommunicable disease outcomes of primary healthcare screening in two rural subdistricts of the Eastern Cape Province, South Africa

**DOI:** 10.4102/phcfm.v9i1.1466

**Published:** 2017-10-31

**Authors:** Paul Rheeder, Angela A. Morris-Paxton, Rose-Marie G. Ewing, Dillon Woods

**Affiliations:** 1 Department of Internal Medicine, Faculty of Health Sciences, University of Pretoria, South Africa; 2 Donald Woods Foundation, Mbashe, South Africa; 3 Nelson Mandela Metropolitan University, South Africa; 4 Donald Woods Foundation, London, United Kingdom

## Abstract

**Background:**

Middle and lower income countries are challenged with a double burden of disease: while still coping with the onslaught of Human Immunodeficiency Virus (HIV) and increasing levels of tuberculosis (TB), there is a considerable increase in the level of noncommunicable diseases (NCDs). The poor are especially disadvantaged and are at an increased risk for NCDs. Adequate healthcare resources for this environment can only be allocated once the extent and exact nature of the problem is determined.

**Aim and setting:**

The aim of this study was to collect demographic and NCD-related data in the poorest community of the poorest province of South Africa in order to determine the extent of the problem and advise on allocation of resources accordingly.

**Methods:**

Data were collected via a household primary health screening process, which included taking anthropometric measurements, blood pressure and blood glucose and referring to clinics for further testing and treatment where necessary.

**Results:**

It was found that the population screened was generally older, consisted of women, and had a high incidence of obesity and hypertension. Of note was the fact that in those without known hypertension, close to 40% of individuals had possible newly diagnosed hypertension. This increased with increase in age and body mass index (BMI). The total prevalence of diabetes was close to 5%, but possible new diabetes was considerably lower at approximately 1%.

**Conclusion:**

In this rural area of the Eastern Cape, South Africa, undiagnosed hypertension is a major concern and renewed efforts at detection and control are warranted.

## Introduction

The threat of noncommunicable disease (NCD) has been recognised globally and the World Health Organization (WHO) has set a target to reduce the overall mortality (from the 2010 baseline) from cardiovascular diseases, cancer, diabetes and chronic respiratory diseases by 25% by 2025.^1^ The latest WHO report on diabetes shows a doubling in the prevalence of diabetes between 1980 and 2014 (from 3.1% to 7.1%, from 4 million to 25 million people). The increase in diabetes prevalence is most pronounced in the lower to middle income countries (LMIC). The percentage of deaths attributed to high blood glucose in those aged 20–69 years in LMIC was 60.5% in men and 45.6% in women.^2^

Regional disparities in diabetes and hypertension prevalence and control also exist. Mills et al. report that in 2010, 31.1% (95% confidence interval [CI]: 30.0% – 32.2%) of the world’s adults had hypertension: 28.5% (27.3% – 29.7%) in high-income countries and 31.5% (30.2% – 32.9%) in LMICs.^3^ An estimated 1.39 billion (1.34–1.44) people had hypertension in 2010: 349 (337–361) million in high-income countries and 1.04 (0.99–1.09) billion in LMICs.^3^ From 2000 to 2010, the age standardised prevalence of hypertension decreased by 2.6% in high-income countries, but increased by 7.7% in LMICs.^3^ During the same period, the proportions of awareness (58.2% vs 67.0%), treatment (44.5% vs 55.6%) and control (17.9% vs 28.4%) increased substantially in high-income countries, whereas awareness (32.3% vs 37.9%) and treatment (24.9% vs 29.0%) increased less, and control (8.4% vs 7.7%) even slightly decreased in LMICs.^3^

Worldwide, the proportion of adults with a body mass index (BMI) of 25 kg/m² or greater increased between 1980 and 2013 from 28.8% (95% CI: 28.4–29.3) to 36.9% (36.3–37.4) in men and from 29.8% (29.3–30.2) to 38.0% (37.5–38.5) in women.^4^ According to the report, the highest rate of obesity and overweight among adults in sub-Saharan Africa is found in South African women, with obesity prevalence at 42%, while the combined rate of both overweight and obesity is 69.3%.^4^ South African men showed a 39% overall prevalence rate, with a prevalence of only 14% obesity.^4^

Sub-Saharan Africa is undergoing a major transition as increased urbanisation and transition from traditional lifestyle to a more Westernised lifestyle is increasing the level of NCDs, while traditional communicable infectious diseases still remain major medical, resource and financial problems for the countries concerned.^5^ Rates of communicable infectious diseases such as HIV, tuberculosis (TB) and malaria are the highest in the world and age standardised cardiovascular diseases (of which higher BMI and hypertension are major risk factors) are up to three times higher in some African countries than in some European countries.^5^ The double burden of both under-nutrition and nutrition-related chronic disease in the region is rising quickly and research has shown that nutrition-related chronic disease is highest among the poor.^6^

Data from the Study on Global Ageing and Adult Health (SAGE), conducted by the WHO, which surveyed more than 35 000 people aged 50 and over in South Africa, China, Ghana, India, Mexico and Russia, found that 78% of South African participants had hypertension.^7^ A study conducted in a rural area of South Africa found that the demographics of rural areas are changing with declining rates of fertility, migration of younger people for work and an increasingly ageing population, requiring different levels of disease management.^8^ As rural populations are increasingly characterised by decreasing number of people of working age and an increase of older people and dependent youth and children, the chronic disease management in this population needs to be prioritised.^9^

South African healthcare has undergone considerable change since 2010 when the Minister of Health, Dr Aaron Motsoaledi, announced the revitalisation of Primary Health Care initiative, involving re-engineering of primary healthcare towards proactive household- and community-focused interventions.^10^ As of writing of this article, the Eastern Cape Province is the poorest province in South Africa with the Wild Coast region ranking as the poorest area within the province.^11,12^ Additionally, the inequality in access to health services is especially disconcerting and has not improved significantly since 1994.^11,12,13^ On a more positive note, the formation of ward-based health outreach teams provided an opportunity to bring healthcare to the door of remote rural people and to gather data on NCD parameters in the community setting. It is in this rural setting that the Donald Woods Foundation (DWF) has implemented a healthcare programme to strengthen the primary healthcare capacity to improve health outcomes. The DWF was established in 2003, with the goal of driving and facilitating rural upliftment and empowerment in the area surrounding Donald Woods’s home of origin and improving communities’ access to health and education.^14^ The result of the Foundation’s initial community consultation and needs analysis was the development and implementation of an extensive health, education and social support programme, including agricultural support, in close partnership with the Department of Health, local communities and traditional leadership.^15^ In mid-2013, through funding received from the Lilly Non Communicable Disease (NCD) Partnership, the DWF embarked on the Health in Every Hut (HiEH) programme in seven clinic catchment areas in the Mbashe Sub-district of the Amathole District, and three clinic catchment areas of the King Sabata Dalindyebo Sub-district of the OR Tambo District in the Eastern Cape Province, South Africa. The purpose of this data collection is to ascertain the full nature of the need for chronic NCD management in order to utilise more effectively both government and non-governmental resources. The aim of this paper is to report on the findings of adult BMI, blood pressure and glucose measurements from the HiEH programme screening.

## Research methods and design

### Study design

This is a cross-sectional descriptive study of initial findings during a household screening survey done in the Mbashe and King Sabata Dalindyebo areas of the Eastern Cape Province.

### Study setting, participant selection and sampling procedure

Identification of geographical areas and households to be targeted in the HiEH intervention was decided in collaboration with the Eastern Cape Department of Health. The Foundation has a framework agreement with the Eastern Cape Provincial Administration, which authorises DWF to work with provincial departments. In addition, a Service Level Agreement, signed by the Superintendent-General of the Eastern Cape Department of Health, covers the services provided by the DWF in support of the department.^15^ Once a clinic catchment area had been delineated and identified for the initiation of screening, a Geographical Information System (GIS) map of the area was generated using Google Earth. Clinic catchment areas often correspond with municipal ward boundaries and typically comprise between 15 and 20 villages, with each village typically having 750 to 1000 inhabitants. Maps of villages were generated for the screening teams to use and to confirm the existence and placement of households. The GPS coordinates of each household were placed onto a customised SQL server, developed for the DWF, along with patient data from screening, clinic referral and Community Health Outreach Worker (CHOW) follow-up visits.

### Data collection

Screening, referrals and follow-up of participants was the responsibility of selected and trained CHOWs. There were 24 Screening CHOWs, and a team of 80 community Embedded CHOWs were responsible for continued clinic referral and follow-up. Screening and Embedded CHOWs were recruited exclusively from the communities in which they resided, through a system of individual applications submitted to clinics, following a broad-brush advocacy campaign across the relevant clinic catchment areas. In the case of each catchment area, 80 to 100 applicants were shortlisted to be assessed. The recruitment process included representation from local communities.

Once the assessment process, conducted over 2–3 days, was completed, the recruitment panel selected 65–80 applicants to attend the first 2 weeks of training, during which time they continued to be assessed against predetermined criteria. These criteria included basic health literacy, ability to learn, enthusiasm, work ethic and punctuality. At the end of the first 2 weeks, 65 applicants were taken forward to complete the 14-week training course, and the assessment continued. At the end of 14 weeks, depending on the need of the catchment area, 24 applicants were selected to be either Screening or Embedded CHOWs for each catchment area. The training that was provided matched the training provided by the Department of Health for their Community Health Workers and the CHOWs also received extensive training on Computer Literacy, Lay Counselling Skills and First Aid. Once trained, CHOWs were placed in the field and were supervised by – on the first level – CHOW Supervisors and – on the second level – CHOW Team Leaders. Each catchment area initially had 24 CHOWs for screening and then eventually 10–12 Embedded CHOWs, a Supervisor and a Team Leader. Screening CHOWs were deployed to visit people in their homes for health screening – bringing primary healthcare to the homesteads of the local population. All members of the household were screened and return visits were made if some members were absent at the time of the first screening. Screening covered seven different areas: hypertension (history and blood pressure measurements), diabetes mellitus (DM; history and blood glucose measurements), maternal and child health, HIV, TB, dementia and identification of orphans and vulnerable children. The latter were identified as children who had lost both parents (orphans), who had no birth certificates, for whom there was no child grant or foster carer grant or who showed signs of neglect or abuse. Any persons with abnormal readings (of either blood pressure or glucose) were referred to an Embedded CHOW for a re-screening or to a clinic for treatment. If anyone screened had a blood pressure value greater than 159/99 or a glucose value greater than 11, they were referred to their local clinic to confirm diagnosis and commence treatment as soon as possible. If the values were lower but still abnormal (for BP 140/90 to 158/98 and for glucose 8–10.9), the individuals were flagged as being at risk and were followed up with repeat measurements by the Embedded CHOWs who were deployed in their own communities to follow up on those people who had high readings or who had been referred from screening to the clinics. Follow-up continued on a regular basis by these Embedded CHOWs.

### Anthropometric measurements

Weight was measured with a Safeway bathroom scale (without shoes, jackets and coats) and height with a tape measure.

### Blood pressure and blood glucose

Prior to June 2015, capillary random glucose was measured with Accu-Chek Active on all clients with a BMI of 25 or more; thereafter On Call EZ glucometres were utilised. The data management programme utilised at that stage did not allow for blood glucose entries if the BMI entered was less than 25; thereafter the system was changed and random blood glucose measurements were routinely done on all people aged 16 years and older to eliminate the possibility of an individual having a normal BMI, but high blood glucose. Blood pressure measurement was done with clients sitting on a chair with arm at the level of the heart after at least 5 min rest with either a Rossmax wrist measurement (2013–2015), thereafter with a Tensoval upper arm measurement (2015 to date). Neither the glucose-measuring devices nor the blood pressure devices were formally validated.

### Data capture

Screening data were captured directly by Screening CHOWs onto a Libre Office database, which was synchronised with the main DWF server daily. This continued from June 2013 to September 2015, after which the data set was amended and data from September 2015 to June 2016 were captured onto MS Excel. Clinic data were captured onto MS Excel, at clinics, by DWF Data Capturers and synchronised with the DWF server on a weekly basis. Follow-up data were largely collected on paper and delivered daily to the DWF monitoring and evaluation team for capturing.

### Data verification

Verification of the data was conducted at the DWF centre, which checked for missing records, anomalies and outliers. Additionally, data were triangulated with written records: duplicate CHOW referral forms were checked against the electronic data for completeness. Google Earth maps were consulted to check that all households in an area had been visited by Screening CHOWs.

Meetings were held with groups of Embedded CHOWs to check electronic follow-up data against written records that CHOWs had kept as backup. Clinic data were checked by a dedicated member of the Clinic Support Team, who checked electronic records against the patients’ clinic files. Verified data for the full period of June 2013 to June 2016 were sent to the Nelson Mandela Metropolitan University where they were linked and imported into a single database.

### Data analysis

Data files collected prior to mid-2015 and thereafter were merged and data quality checked and errors corrected where possible. In the case of errors not remediable or values highly unlikely, these were changed to missing. For the sake of NCD evaluation, the analysis was restricted to adults aged 18 years or older and adults who were HIV positive, pregnant or had active tuberculosis were excluded from the analyses. Diabetes and hypertension were defined if a person reported the condition or if they were on medication for the condition at the time of questioning. Data were analysed in STATA version 14^16^ and R version 3.3.1 (2016-06-21).^17^ Summaries with descriptive statistics are given and group comparisons made with parametric tests for continuous data and chi-square tests for categorical data. The trend test used performs the nonparametric test for trend across ordered groups developed by Cuzick (1985),^18^ which is an extension of the Wilcoxon rank-sum test. Glucose categories were classified using cut points recommended by the Society for Endocrinology, Metabolism and Diabetes South Africa diabetes guideline^19^ for diabetes testing and not the cut points used for screening. This was done in order to make comparisons with other studies possible. Age categories were in accordance with the South African National Health and Nutrition Examination Survey.^20^ BMI categories were done according to WHO definitions.^21^ Blood pressure categories were classified using the South African Hypertension guidelines.^22^ Age standardisation was done using the WHO world population^23^ with age categories restricted to 20–99 in men and women separately and weights calculated for the age strata between 20 and 99 years.

### Ethical consideration

The programme and evaluation were approved by the University of Pretoria Faculty of Health Sciences Research Ethics Committee: The monitoring and evaluation of the Donald Woods Foundation Health in Every Hut programme (341/2016).

## Results

Of the 17 350 people with data available for analysis, 66% were women and the mean age of men and women was 44 years. Results regarding BMI, glucose and blood pressure can be seen in Table 1.

**TABLE 1:** Characteristics of the study population, mean (standard deviation).

Variable	[ALL] *N* = 17 350	Female *N* = 11 491	Male *N* = 5859	*P*	*N*
Age	43.5 (20.1)	44.5 (19.8)	41.4 (20.5)	< 0.001	17 350
BMI	25.8 (5.9)	27.1 (6.2)	23.2 (4.0)	< 0.001	17 111
Glucose	6.1 (2.5)	6.1 (2.6)	6.0 (2.3)	0.165	8499
Systolic BP	130.6 (23.5)	130.7 (24.0)	130.4 (22.5)	0.498	17 236
Diastolic BP	85.9 (17.6)	86.4 (18.3)	85.0 (16.2)	< 0.001	17 233

BP, blood pressure; BMI, body mass index; *N*, number; *P, p*-value.

**FIGURE 1:**
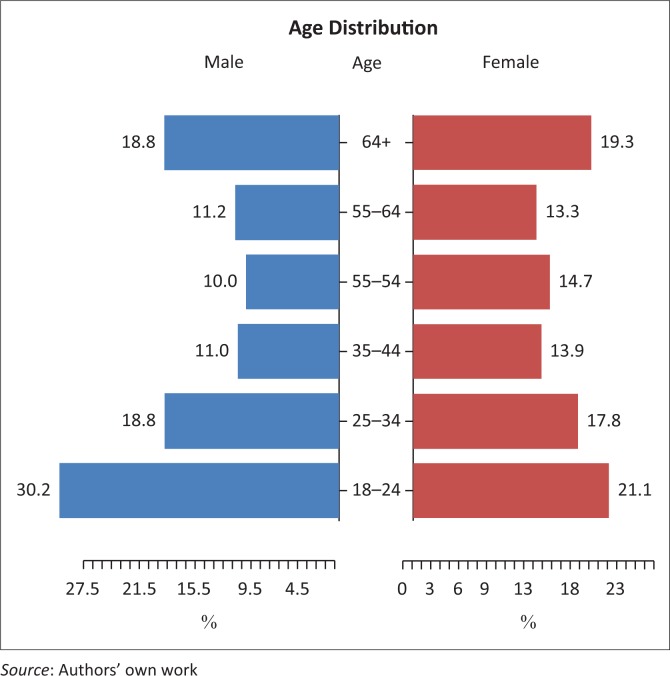
Age distribution of study population.

Despite the mean age reflecting 44.5 years, the standard deviation is large and the actual age distribution as given in Figure 1 reflects the picture of a population that is on the one hand ageing and on the other under 25, with fewer people of working age in-between. There was a statistically significant difference in blood pressure between men and women (Table 2); however, the differences in proportions were small.

**TABLE 2:** Blood pressure categories among men and women.

Gender	BP category					Total *N* (%)
	Normal *N* (%)	High normal *N* (%)	Stage 1 HT *N* (%)	Stage 2 HT *N* (%)	Stage 3 HT *N* (%)	
Female	4079 (42.3)	1647 (17.1)	2315 (24)	904 (9.4)	687 (7.1)	9632 (100)
Male	2248 (41.9)	1032 (19.2)	1267 (23.6)	476 (8.9)	347 (6.5)	5370 (100)
**Total**	**6327 (42.2)**	**2679 (17.9)**	**3582 (23.8)**	**1380 (9.2)**	**1034 (6.9)**	**15 002 (100)**

*N*, number; HT, hypertension; *P*-value, *p* = 0.015.

Blood pressure levels increased across age categories, with 50% of people 55 years or older having hypertension (*P* < 0.001 for trend). Similarly, blood pressure levels also increased across BMI categories with 52% of people with BMI 30 or above having hypertension (*P* < 0.001 for trend). Age standardised prevalence of new hypertension for men was 41% and that for women was 43%. Total hypertension (new plus known HT)-standardised prevalence was 43% for men and 50% for women.

The differences in glucose were not statistically different between men and women (Table 3). The age standardised prevalence of possible new diabetes (>11 mmol/l) was 0.8% in men and 1.3% in women. Glucose levels increased across age categories with 14% of people 55 years and older having glucose values of 7.8 mmol/L and above (*P* < 0.001 for trend). As expected, glucose levels also increased across BMI categories with 9.1% of those with a BMI of 30 and above having glucose values of 7.8 mmol/L and above (*P* < 0.001 for trend). The standardised prevalence of total diabetes (new and known DM) was 5% in men and 5.5% in women.

**TABLE 3:** Glucose categories among men and women.

Gender	Glucose category *N* (%)			Total
	< 7.8	7.8–11	> 11 mmol/L	
Female	5944 (92.9)	368 (5.8)	84 (1.3)	6396 (100)
Male	1684 (92.6)	118 (6.5)	16 (0.9)	1818 (100)
**Total**	**7628 (92.9)**	**486 (5.9)**	**100 (1.2)**	**8214 (100)**

*N*, number; *P*-value, *p* = 0.174.

## Discussion

The data collected by the CHOWs in the HiEH programme provide a unique snapshot of the challenge of NCDs in the rural Eastern Cape. The re-engineering of healthcare with the focus on teams operating in the district provides valuable data on the health status in the communities in which they serve. This study was conducted in a very challenging environment necessitating healthcare workers to travel large distances over inaccessible terrain to reach participants in their homes. The majority of participants were women (66%) with a mean age of 44 years in the study population. The age pyramid shows that in this adult population the largest proportions were in the 18- to 24-year and ≥ 64-year age groups reflecting the young unemployed and the elderly.

The most important finding in this screening survey was the alarmingly high prevalence of hypertension (43% in men and 50% in women, age standardised). This is in agreement with other data from South Africa.^7^ In a study on hypertension in nine LMIC, Irazola et al. reported on age-and sex-standardised prevalence rates of hypertension among men and women 35–74 years of age in selected communities.^24^ Rates of hypertension were found to be 49.9% (95% CI: 42.3% – 57.4%) in Kenya, 54.9% (95% CI: 51.3% – 58.4%) in South Africa, 52.5% (95% CI: 50.1% – 54.8%) in China, 32.5% (95% CI: 31.7% – 33.3%) in India, 42.3% (95% CI: 40.4% – 44.2%) in Pakistan, 45.4% (95% CI: 43.6% – 47.2%) in Argentina, 39.9% (95% CI: 37.8% – 42.1%) in Chile, 19.2% (95% CI: 17.8% – 20.5%) in Peru and 44.1% (95% CI: 41.6% – 46.6%) in Uruguay.^24^ The proportion of awareness varied from 33.5% in India to 69.0% in Peru, the proportion of treatment among those who were aware of their hypertension varied from 70.8% in South Africa to 93.3% in Pakistan, and the proportion of blood pressure control varied from 5.3% in China to 45.9% in Peru.^24^

In the Eastern Cape study population, the prevalence of high glucose levels warranting further investigation was low. The Multi-ethnic Study of Pre-Diabetes and Diabetes in LMIC reported that diabetes prevalence was higher in South Africa (13.8%) than Peru (9.8%) but less than South Asia (19%).^25^ The South Africa data came from the Cardiovascular Risk in Black South Africans study (a cross-sectional study of 1099 men and women, aged 25–74 years, from predominately Black residential areas of Langa, Guguletu, Crossroads, Nyanga and Khayelitsha in Cape Town recruited between 2008 and 2009). The International Diabetes Federation cites that South Africa had a DM prevalence of 7% (2.28 million cases) in 2015.^26^

The prevalence of obesity was significantly higher in women than in men (age standardised prevalence for men was 7.7% and that for women was 32%). This is lower than that reported in the Global Burden of Disease study (42% obesity in women and 13.5% in men).^4^ This high prevalence of hypertension in this study is disconcerting given that the prevalence of hypertension is generally found to be lower in rural areas compared with urban areas,^27,28^ although Mollentze found that in the Free State there was no difference between rural and urban participants.^29^ The rural, urban and migrant differences in NCD risk factors in middle-income countries were evaluated using the WHO-SAGE data.^30^ That study compared NCD risk factor prevalence in urban, rural and migrant populations in China, Ghana, India, Mexico, Russia and South Africa. Overweight, raised waist circumference and diagnosed diabetes were higher in urban groups (relative risks using rural as reference group, 1.19 [1.04–1.35], 1.24 [1.07–1.42], and 1.69 [1.15–2.47], respectively).^30^ Migrant groups in Mexico had significantly higher prevalence of hypertension than rural groups (1.46 [0.97–1.97]), whereas in South Africa this was reversed (0.71 [0.34–1.12]).^30^ In contrast, diagnosed diabetes showed a very consistent association across all the countries studied with significantly higher prevalence of diagnosed diabetes in migrant and urban groups in the pooled analyses (1.60 [1.04–2.43]; 1.69 [1.15–2.47]).^30^ This association was also significant for urban groups in the country-specific analyses for China, India and South Africa (2.18 [1.35–3.47]; 1.44 [0.95–2.18]; 3.48 [1.64–7.09]) and for migrant groups in China and Ghana (1.86 [1.05–3.25]; 5.15 [1.89–13.50]).^30^

Regarding hypertension, the WHO has set the target for 2025 (against a baseline of 2010) as a 25% relative reduction in the prevalence of raised blood pressure or to contain the prevalence of raised blood pressure, according to national circumstances.^1^ The South African Government is taking the challenge of NCDs seriously and has defined three primary approaches required to control these diseases.^10^ The first is to focus on health promotion and primary prevention at the individual and community levels, the second is to improve NCD control through health systems strengthening, and the last is to expand surveillance of NCDs and associated risk factors and conduct research on these subjects.^10^ This together with the mentioned re-engineering of healthcare will expectedly translate into improved detection and management of NCDs with special emphasis on hypertension.^10,31^

### Wider implications for future cross-sectoral community access and service delivery

The DWF has demonstrated a model for community access to primary healthcare in a deeply rural, widely dispersed population, involving health screening for 50 000 people over 1000 square kilometres. The philosophy and primary driver in the concept design was to place the elderly person living furthest from the road as a person of equal value and inclusion as the person living next to the clinic. Most projects combined with environmental geography and logistics mean that homesteads near dirt or tarred roads have much more access to government services, staff, logistics and infrastructure of any kind, especially health. This project was designed so everyone, no matter how disadvantaged or remote they were in any way, gained the same access and level of support as the person living next to the clinic.

On each day of screening, the data were synchronised overnight, to ensure that each person was recorded as a living, valuable individual, while their data and requirements were available for sharing with local clinics. Clinic staff then followed through the process and the DWF followed up on patients at home so that they were not neglected, left behind or falling through the net. Some large-scale, paper-based screening projects have gathered data which are sitting in boxes, unread, thereby placing no present, ongoing and live value to the individual screened, unlike the above model.

The logistical success of this programme and its design for equity in access for all rural people demonstrate through evidence and data to show how all homesteads, including the most remote homesteads, can benefit from access to government services, staff, logistics and infrastructure that go beyond health.

The above model has been developed and piloted to show the degree of logistical and human resource capacity required to bring each person into a position of enhanced value in their community and to connect them with their rights and needs. These rights and needs can give rise to health, education, social development, agricultural and other programmes, local initiatives and synchronisation of joint initiatives, particularly in the areas of access to markets, job creation and small business start-ups.

Should government departments be able to pool their resources to provide a combined approach in rural areas, then engagement with the entire rural population of the country would produce a significant return on this investment through a concrete reduction in poverty and income generation in areas where it is required the most. This study was conducted over a 3-year period, from June 2013, and as such constitutes a pilot study of the re-engineering of primary healthcare methodology described above.

## Limitations

A limitation of the study was the fact that two different blood pressure devices were used, one of which was a wrist device. We do not have method comparison data available for these two devices. We used capillary glucose measurements, which are not ideal but capillary glucose measurements are often used in population studies.^32^ The measurement devices were also not validated. A second limitation was that the data capture system was changed in 2015 for reasons beyond the control of DWF; however, the second system in use allowed for the capturing of all blood glucose measurements, whereas the previous system prior to 2015 had not.

## Recommendations

It is clear from this study that community workers are able to detect undiagnosed hypertension and evaluation of referral. Treatment of these individuals will be key in determining if the intervention leads to better control and outcomes.

## Conclusion

In this rural area of the Eastern Cape, South Africa, undiagnosed hypertension is a major concern and renewed efforts at detection and control are warranted. Undiagnosed possible diabetes appears to be less of a problem in this population.
